# Nasal Levels of Antimicrobial Peptides in Allergic Asthma Patients and Healthy Controls: Differences and Effect of a Short 1,25(OH)_2_ Vitamin D3 Treatment

**DOI:** 10.1371/journal.pone.0140986

**Published:** 2015-11-06

**Authors:** Willemien Thijs, Kirsten Janssen, Annemarie M. van Schadewijk, Socrates E. Papapoulos, Saskia le Cessie, Saskia Middeldorp, Christian F. Melissant, Klaus F. Rabe, Pieter S. Hiemstra

**Affiliations:** 1 Department of Pulmonology, Leiden University Medical Center, Leiden, The Netherlands; 2 Department of Endocrinology, Leiden University Medical Center, Leiden, The Netherlands; 3 Department of Clinical Epidemiology and Department of Medical Statistics, Leiden University Medical Center, Leiden, The Netherlands; 4 Department of Vascular Medicine, Academic Medical Center, Amsterdam, The Netherlands; 5 Department of Pulmonology, Spaarne Hospital, Hoofddorp, The Netherlands; 6 Department of Pulmonology and Thoracic Surgery, Lungen Clinic Grosshansdorf, Grosshansdorf, Germany; University Hospital Freiburg, GERMANY

## Abstract

**Background:**

Allergy is often accompanied by infections and lower levels of antimicrobial peptides (AMPs). Vitamin D has been shown to increase expression of selected AMPs. In this study we investigated whether antimicrobial peptide levels in nasal secretions of allergic asthma patients are lower than in healthy controls, and whether administration of the active form of vitamin D (1,25(OH)_2_D3) affects these antimicrobial peptide levels.

**Methods:**

The levels of antimicrobial peptides in nasal secretions were compared between 19 allergic asthma patients and 23 healthy controls. The effect of seven days daily oral treatment with 2 μg 1,25(OH)_2_D3 on antimicrobial peptides in nasal secretions was assessed in a placebo-controlled cross-over clinical study.

**Results:**

Levels of neutrophil α-defensins (human neutrophil peptides 1–3; HNP1-3) and lipocalin 2 (LCN2; also known as NGAL) were significantly lower in asthmatics, but no differences in LL-37 and SLPI were detected. Treatment with a short-term 1,25(OH)_2_D3 caused a small increase in HNP1-3, but not when the asthma and control groups were analyzed separately. LL-37, LCN2 and SLPI did not change after treatment with 1,25(OH)_2_D3.

**Conclusion:**

Levels of the antimicrobial peptides HNP1-3 and LCN2 are lower in nasal secretions in asthmatics and are not substantially affected by a short-term treatment with active vitamin D.

## Introduction

Exacerbations in asthma are associated with increased airway inflammation, decreased lung function and increased symptoms, and are frequently accompanied by respiratory infections[1,2]. It has been shown that allergic airways disease is accompanied by increased susceptibility to infections[3–5]. This may in part be explained by the observation that allergic inflammation decreases local host defense against infections by reducing the expression of antimicrobial peptides and proteins (AMPs), which is mediated at least in part by Th2 cytokines[6,7].These AMPs form an essential element of innate immunity in most multicellular organisms, are mainly produced by neutrophils and epithelial cells, and kill a wide range of bacteria, fungi, viruses and microbes[8]. Various studies revealed deficiencies of selected AMPs in airway secretions of patients with allergic rhinitis, sinusitis and asthma[6,9,10]. The role of AMPs in allergic asthma is incompletely understood, but in view of the diverse role of these molecules in regulating host defense, immunity and wound repair they are likely important players in allergic asthma.

Vitamin D has been identified as a key regulator of production of AMPs such as the cathelicidin (hCAP18/LL-37) and the neutrophil gelatinase-associated lipocalin (LCN2) in epithelial cells[11,12]. Allergen challenge in allergic asthmatics has been shown to cause an increase in hCAP18/LL-37 and inflammatory markers in bronchoalveolar lavage, which was accompanied by an increase in vitamin D[13]. Furthermore, vitamin D administration has been shown to increase cathelicidin expression in patients with atopic dermatitis and in neonates[14,15].Collectively, these data suggest that allergic inflammation contributes to impaired host defense against infections, and that vitamin D could improve this by stimulating AMP production. However, there is limited data on AMPs expression in allergic asthmatic patients and to our knowledge there are no data on the effect of vitamin D treatment on AMPs in asthma patients to support a role for vitamin D in reducing exacerbations.

Our study had two objectives: First, we examined the expression of AMP levels in nasal secretions from patients with allergic asthma and in healthy controls in a case control design. To this end we focused on AMPs that are either only expressed in neutrophils (human neutrophil peptides [HNP]1–3; neutrophil α -defensins) or in epithelial cells (secretory leukocyte proteinase inhibitor; SLPI), or in both cell types (LL-37 and LCN2) and compared the levels of these AMP in asthma patients and healthy controls. Secondly, we assessed if vitamin D administration increased AMP levels in both asthma patients and healthy controls in a placebo-controlled cross-over design. Since the main circulating form of vitamin D, 25(OH)D3, requires local conversion to the active form (1,25(OH)_2_D3) by CYP27B1 and since expression of CYP27B1 is regulated by microbial exposure and inflammation[16], we administered the active form[17].

## Material and Methods

### Participants

For the first case-control part of our study we aimed to include twenty allergic intermittent asthmatic patients and twenty healthy controls. Participants were recruited by the Department of Pulmonology of the Leiden University Medical Center (LUMC) by advertisement in the Leiden area of the Netherlands. The sample size was based on repeatability of AMP immunoassays from our laboratory and on the data on hCAP18/LL-37 levels in sputum from asthmatics[10]. Exclusion criteria for all participants (asthmatics and healthy controls) were use of vitamin D supplements or antihistamines, smoking or ex-smoking with more than 5 pack-years, pregnancy and a recent (≤2 weeks) upper respiratory tract infection or other relevant diseases. Furthermore participants under 18 and above 45 years were excluded as well as participants who used inhaled corticosteroids during the last 4 weeks or oral corticosteroids within 3 months before the study.

The inclusion criteria for the twenty patients with allergic intermittent asthma were a history of episodic chest tightness or wheezing (but no daily symptoms or symptoms at night), PC_20_ methacholine less than 9.6 mg/ml and atopy, as determined by a positive skin prick test result (≥3mm wheal) to 1 or more of 10 common aeroallergen extracts (HAL, Haarlem, the Netherlands). Furthermore their baseline FEV_1_ had to be above 70% of predicted[18]. The inclusion criteria for the healthy controls were no history of episodic chest tightness or wheezing, PC_20_ methacholine more than 9.6 mg/ml, a negative skin prick test, and their baseline FEV_1_ should be more than 80% of predicted.

All participants, asthmatics and healthy controls, were included in the intervention part with 1,25(OH)_2_D3 of our study. The protocol was approved by the institutional review board for human studies and before entering the study, the participants gave their written informed consent.

### Study design

**Fig 1:**
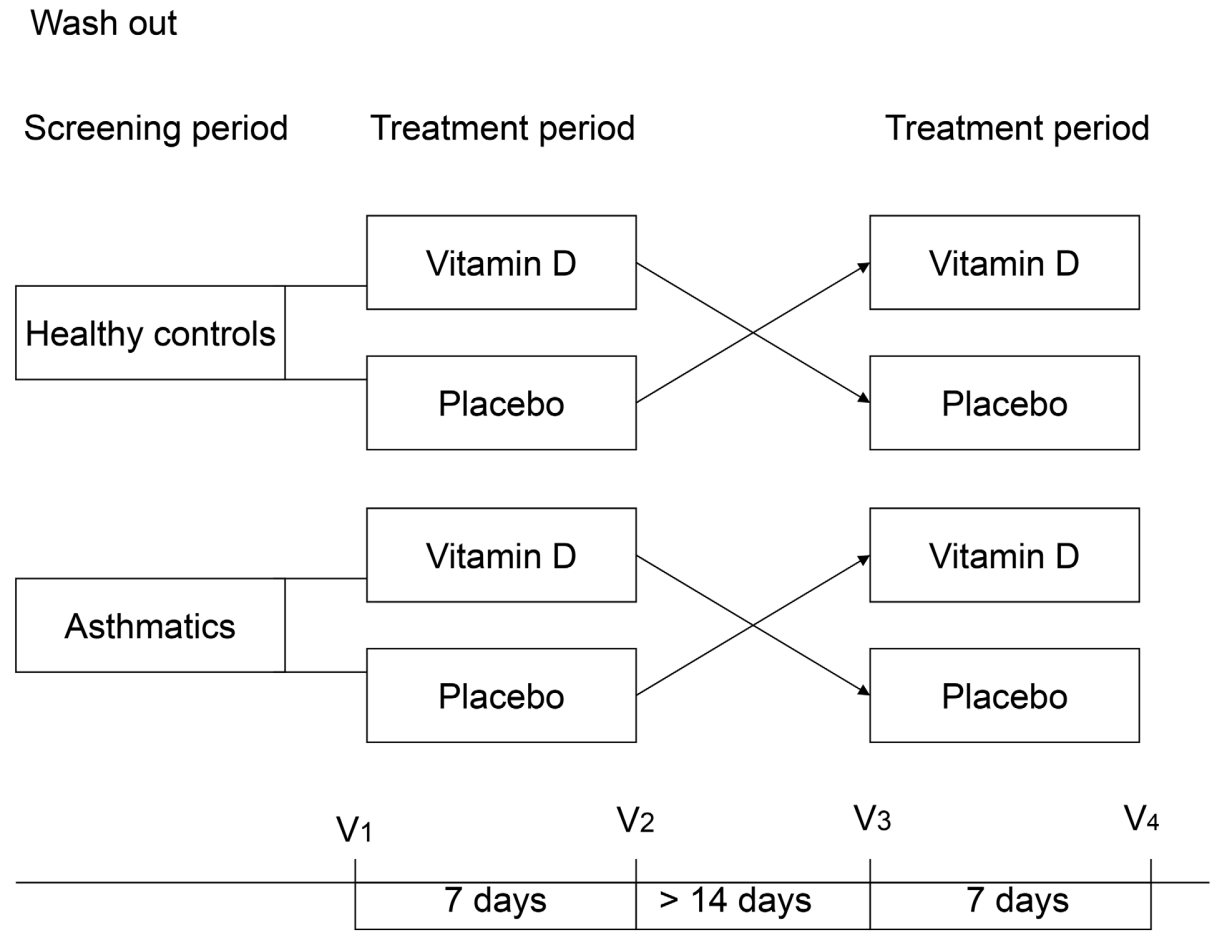
Single-blind, placebo-controlled cross-over design of the present study.

The levels of AMPs in nasal secretions at baseline in patients with mild-to-moderate allergic asthma were compared to non-allergic controls in an unpaired comparison of participants stratified by disease. The effect of the intervention with 1,25(OH)_2_D3 was studied in a double-blind, placebo-controlled cross-over design (Fig 1) that was similar in asthmatics and healthy controls. Treatment order was determined by randomization. Samples were collected and measurements performed on 7 separate visits. At the inclusion visit, medical history was taken, atopy was determined and provocative concentration causing a 20% fall in FEV_1_ was measured (PC20 metacholine). In four visits before and after 7 days of 1,25(OH)_2_D3 or placebo, nasal secretions and venous blood was collected. An additional control visit was made halfway through each of the two treatment weeks, and venous blood was taken to assess calcium levels as a safety measure for the treatment. Between the intervention periods, a two week wash out period was scheduled (Fig 1).

### Intervention

All participants received tablets of 2 microgram 1,25(OH)_2_D3 or placebo once daily during seven days[17,19].

### Exhaled nitric oxide

Exhaled nitric oxide (eNO) was measured using a portable analyzer, the NIOX MINO (Aerocrine AB, Solna, Sweden). Participants performed three 10-seconds slow steady exhalation maneuvers, and the mean was used to calculate the levels of eNO.

### Spirometry

Flow-volume curves were recorded by pneumotachograph to obtain the forced expiratory volume in 1 second (FEV_1_) and the forced vital capacity (FVC).

### Nasal secretions

Nasal secretions were collected by vacuum-aided suction[20]. Manipulation with a narrow-tipped vacuum device was used to mildly stimulate nasal secretions. This permits the collection of undiluted whole nasal fluid without the unpredictable effects of dilution in saline or other lavage fluid. Gentle manipulation of a narrow rubber-tipped vacuum device inside the nasal passageways stimulates nasal secretions. Secretions were stored at -80°C for longer-term storage. Nasal secretions were homogenized by brief (3 times 10-second) microtip sonication.

### Laboratory analyses

The nasal secretions were analyzed for the presence of antimicrobial peptides and proteins, IL-8 (as a measurement for inflammation) and albumin (as a measurement for vascular leakage). In addition, hCAP18/LL-37 levels were analyzed in plasma. Commercially available ELISA kits were used to detect LCN2 (Bioporto), hCAP18/LL-37 and HNP1-3 (Hycult Biotech), and IL-8 (Sanquin). The SLPI ELISA was developed in our laboratory at the Leiden University Medical Center[21]. The absorbance was measured at 450 nm using a Microplate reader (model 680; Bio-Rad, Hercules, CA) and Microplate Manager software (version 5.2.1, Bio-Rad). The lower limits of detection were: LCN2 10 pg/ml; SLPI 10 ng/ml; HNP1-3 150 ng/ml; LL-37 20 ng/ml and IL-8 200 pg/ml. Albumin levels were determined using nephelometry (Siemens BN Prospec) with a lower limit of detection of 17 μg/ml. Inter and intra-assay variability for all assays was < 10%.

Serum 1,25(OH)_2_D3 and 25(OH)D3 were assessed at the central clinical chemistry laboratory of the LUMC. Quantification of the 25(OH)D3 concentration in the serum was done using a DiaSorin ^125^I RIA Kit (DIASORIN, INC.). Quantification of the 1,25-(OH)D3 concentration in the serum was done using a DiaSorin ^125^I RIA Kit (DIASORIN, INC.) preceded by extraction and column separation.

### Data and statistical analyses

Levels of antimicrobial peptides in mild to moderate asthma versus healthy controls were compared using a Mann-Whitney test. The measurements before and after placebo treatment en the measurements before 1,25(OH)_2_D3 treatment were used to calculate the mean baseline levels for each participant because these measures are not affected by the 1,25(OH)_2_D3 treatment (Fig 1). Spearman rank coefficient was used to explore the associations between the AMPs and 25(OH)D3. hCAP18/LL-37 in plasma was log-transformed and a Pearson correlation was used to calculate the association with 25(OH)D3. Pearson correlation was used to explore the relation between albumin, 25(OH)D3, 1,25(OH)_2_D3, AMPs and IL-8. The effect of 1,25(OH)_2_D3 treatment was assessed by calculating the change in outcome measures before and after a treatment or placebo. In this analysis only those patients were included in whom before and after treatment samples were available for a 1,25(OH)_2_D3 or placebo treatment period. The change after 1,25(OH)_2_D3 treatment in the whole group was compared to the change after placebo treatment within subjects using the Wilcoxon signed rank test. Statistical analyses were performed with SPSS 20.0 software (SPSS Inc., Chicago, IL). Statistical significance was inferred at p < 0.05.

## Results

We included a total of 42 participants from the end of 2008 till the spring of 2010 (19 atopic asthma patients and 23 healthy controls). One healthy participant only completed the first treatment period. Participant characteristics are shown in Table 1, and there were no significant differences between both groups. The mean age of the atopic asthma patients was 27.8 (IQR 22–29) years; the healthy controls were slightly younger and their average age was 23.4 (IQR 20–24) years. There was substantial variability in baseline serum 25(OH)D3 levels (S1 Fig). Only one (asthmatic) participant showed a severe deficiency (<30 nM). The BMI of the asthma patients was 22.9 (IQR 21.6–24.4) and for the healthy controls this was 21.5 (IQR 20.1–22.7). Neither the difference in age nor BMI were statistically significant.

**Table 1:** Clinical characteristics of the study population according to asthma status.

	Atopic asthma (n = 19)				Healthy controls (n = 23)				
	Mean	Range	IQR	SD	Mean	Range	IQR	SD	p-value
Age (years)	27.8	19–45	22–29	8.1	23.4	18–45	20–24	6.3	0.06
Sex (male %)	26.3	NA	NA	NA	26.1	NA	NA	NA	0.99
BMI (kg/m^2^)	23	19–26	22–24	2.3	22	18–27	20–23	2.1	> 0.05
FEV_1_ (%)	97	79–127	88–107	13.3	102	85–121	93.2–110	10.8	0.37
25(OH)D3 (nmol/L)	65	10–127	45–91	31	55	23–113	35–71	23.2	0.22
Exhaled nitric oxide (ppb)	54	19–135	28–71	28	14	7–25	10–17	5	< 0.00

BMI: Body mass index; IQR: Interquartile range; SD standard deviation, NA: not applicable; FEV_1_: percent predicted of FEV_1_, ppb parts per billion p-values for differences between groups

When comparing the difference in the AMP and IL-8 levels in nasal secretions between atopic asthmatics and healthy controls, HNP1-3, LCN2 and IL-8 were found to be significantly lower in atopic asthmatics (Table 2). LL-37, SLPI and albumin levels did not differ significantly between atopic asthmatics and healthy controls.

**Table 2:** Median baseline AMPs, IL-8 and albumin in nasal secretions according to asthma status.

	Asthma mean (IQR)	Healthy control mean (IQR)	significance
HNP 1–3 (ng/ml)	5194 (1991, 5730)	10618 (3381, 15363)	0.02*
LL-37 (ng/ml)	99 (42, 155)	182 (61, 268)	0.12
LCN2 (ng/ml)	2272 (1104, 3143)	4303 (2150, 6186)	0.01*
SLPI (μg/ml)	674 (112, 398)	1008 (104,471)	0.79
IL-8 (ng/ml)	2 (0.5, 4)	7 (2, 13)	<0.05*
Albumin (μg/ml)	653 (271, 889)	326 (146, 563)	0.088

*statistical significant < 0.05

No correlation was found in the total group of participants between the baseline levels of antimicrobial peptides and IL-8 in nasal secretion with serum 25(OH)D3. At baseline albumin levels in nasal secretion were not correlated with serum 25(OH)D3 or 1,25(OH)_2_D3 in the whole group and also not for asthma patients and healthy controls apart. However, there was a significant correlation between albumin levels and HNP 1–3 (r = 0.509 p = 0.013), IL-8 (r = 0.643 p = 0.001) and hCAP18/LL-37 (r = 0.460 p = 0.029) in healthy controls. In asthma patients albumin was not correlated with any of the AMPs or IL-8.

**Fig 2:**
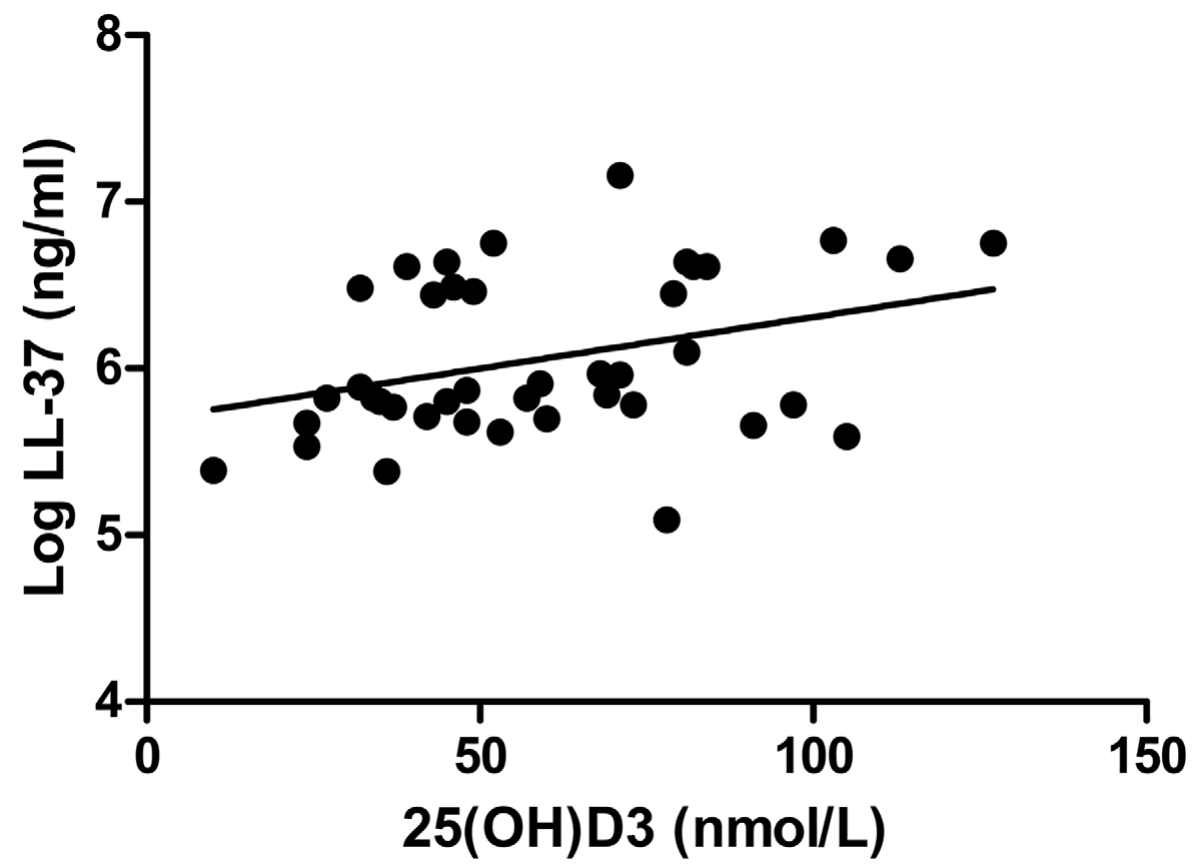
Correlation between 25(OH)D3 in serum and LL-37 in plasma. Log-transformed LL-37 levels were significantly correlated to 25(OH)D3 levels (Pearson correlation 0.343, p = 0.028).

hCAP18/LL-37 plasma levels (log transformed because of their non-normal distribution) and 25(OH)D3 at baseline were significantly correlated (Pearson correlation 0.343, p = 0.028) (Fig 2) for the whole group; for healthy controls and asthmatics analyzed separately there was no difference.

To evaluate the effect of vitamin D treatment, participants were treated for one week with 1,25(OH)_2_D3. This treatment duration was selected based on the kinetics of vitamin D-induced expression of antimicrobial peptides in *in vitro* studies[11,12]. Furthermore, a study by Xystrakis *et al* that showed that vitamin D3 treatment of steroid resistant asthma patients for 7 days enhanced *in vitro* responsiveness to dexamethasone of cultured CD4^+^ T cells[22]. Samples were available for most patients to evaluate the effect of treatment: 39/42 in the placebo period, and 38/42 in the 1,25(OH)_2_D3 treatment period. Serum levels of 1,25(OH)_2_D3 increased significantly after intervention (mean difference 86 pg/ml (95% CI 55–116)), but four participants did not show such an increase of 1,25(OH)_2_D3. All patients had a control visit halfway through the treatment period during which serum calcium and creatinine levels were checked. None of the patients had abnormal levels during treatment and none of the participants reported side affects after treatment. The effect of 1,25(OH)_2_D3 treatment on serum levels of 1,25(OH)_2_D3 for asthma and healthy participants are shown in Table 3.

**Table 3:** Effect of 1,25(OH)_2_D3 treatment on serum 1,25(OH)_2_D3, and nasal AMPs and IL-8 levels according to asthma status.

	Asthma patients (change^#^)			Healthy controls (change^#^)		
	Placebo	1,25(OH)_2_D3	p-value	Placebo	1,25(OH)_2_D3	p-value
1,25(OH)_2_D3 (ng/ml)	6	86	0.004	3	97	0.000
HNP1-3 (ng/ml)	-44	1392	0.40	2875	1967	0.07
LL-37 (ng/ml)	-30	5	0.39	-1	22	0.30
LCN2 (ng/ml)	217	448	0.87	635	448	0.40
SLPI (μg/ml)	-10	-5	0.73	19	30	0.23
IL-8 (ng/ml)	-0.1	0	0.17	0.1	1.7	<0.05

^#^ change denotes the median difference between the value on day 7 and day 1 of treatment period

**Fig 3:**
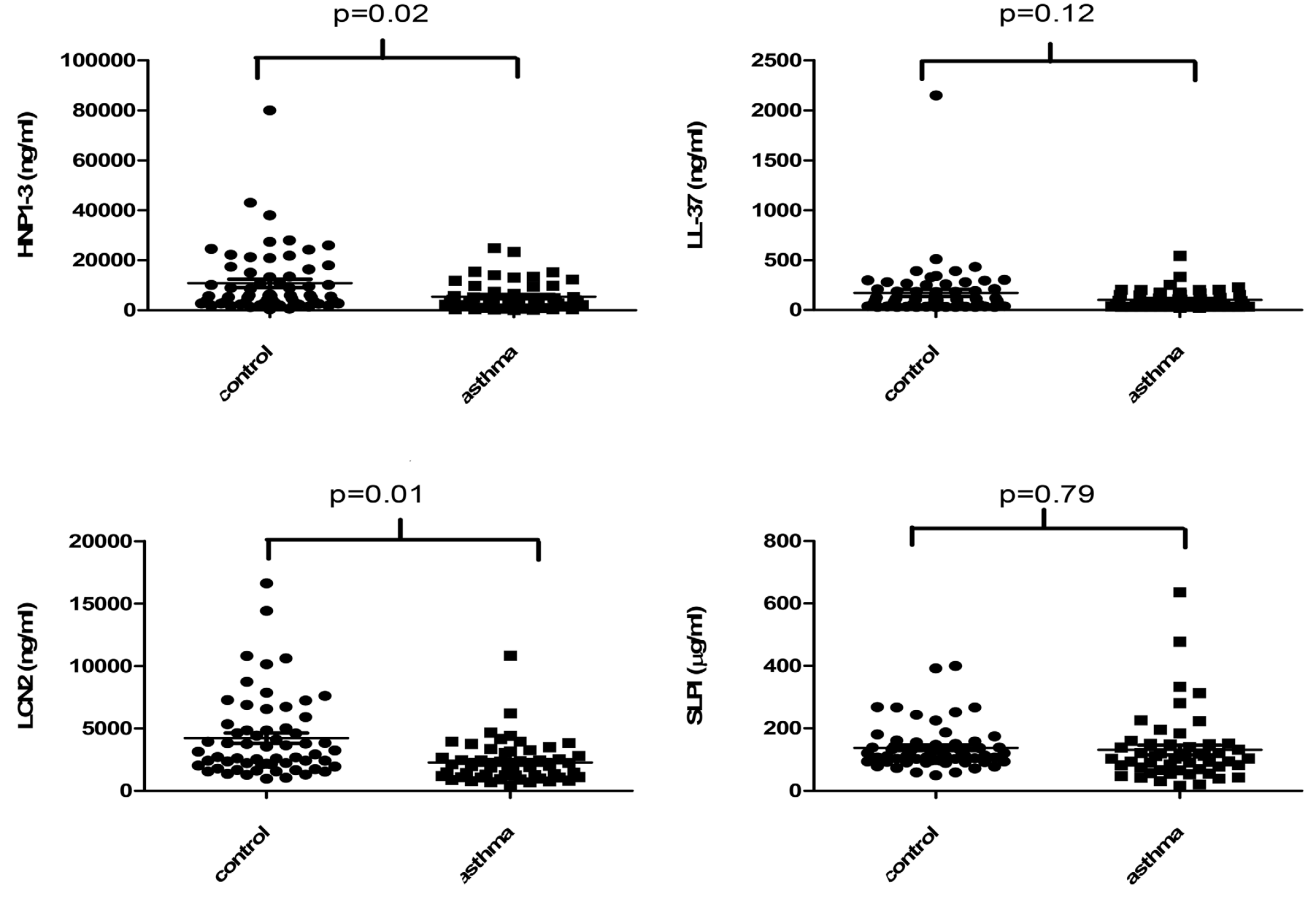
Baseline AMPs, in nasal secretions. HNP1-3, LL-37, LCN2 and SLPI in nasal secretions from atopic asthma patients and healthy controls.

The effects of 1,25(OH)_2_D3 treatment on antimicrobial peptides and IL-8 in nasal secretions for asthma and healthy participants are shown in Table 3 and Fig 3. In healthy controls, 1,25(OH)_2_D3 treatment caused a small but significant increase in IL-8 levels. In the group of asthmatics, 1,25(OH)_2_D3 treatment was associated with an non-significant increase in HNP1-3. In healthy controls, the reverse was observed and there was a trend (p = 0.07) for a stronger effect of placebo treatment than 1,25(OH)_2_D3 treatment on increasing HNP1-3 levels.

**Fig 4:**
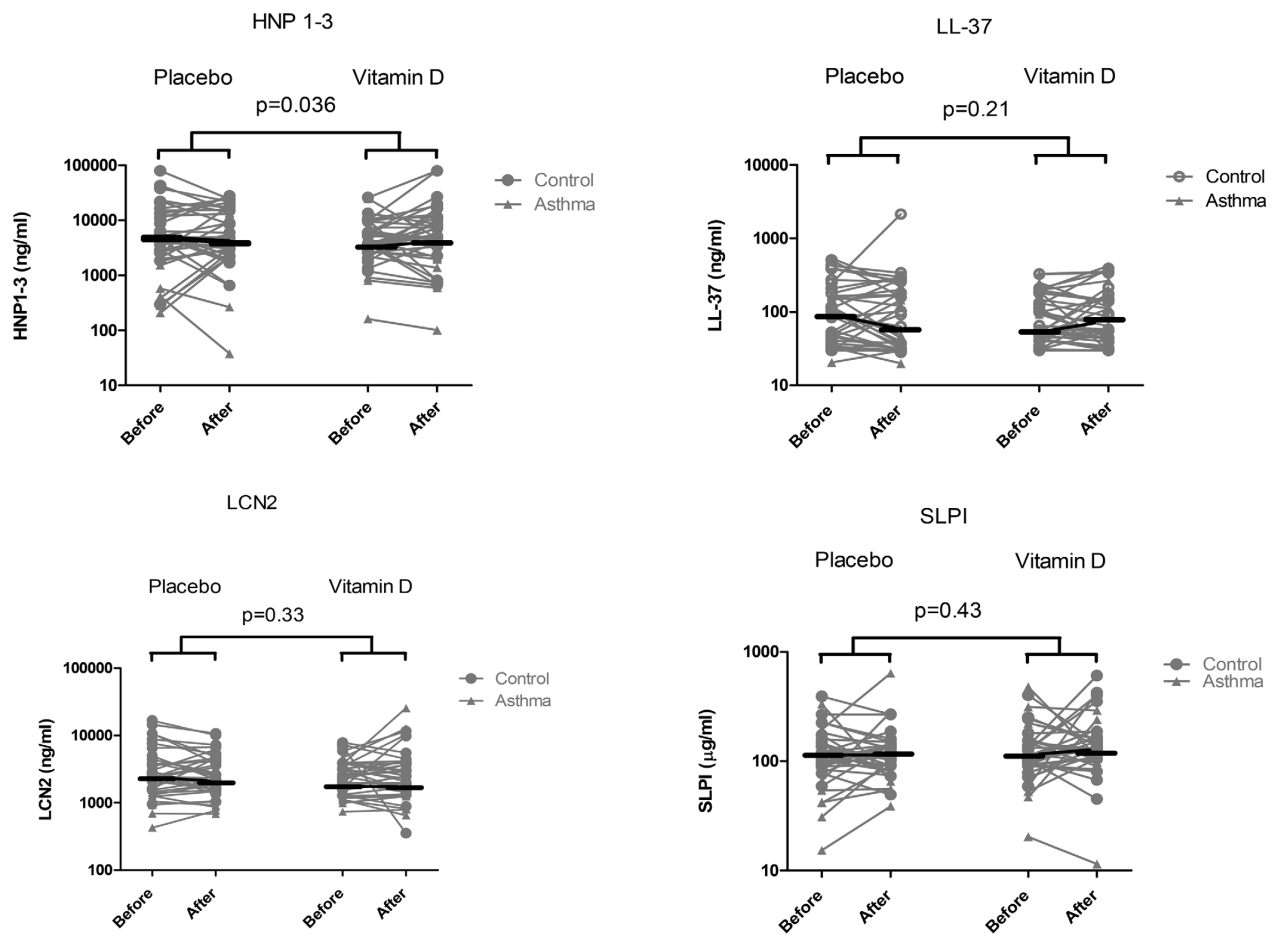
Effect of 1,25(OH)_2_D3 treatment on levels of AMPs in nasal secretions. Change in levels of HNP1-3, LL-37, LCN2 and SLPI in nasal secretions from asthma patients and controls during placebo or 1,25(OH)_2_D3 treatment. The horizontal bars represent the median AMP level before and after treatment.

We next analyzed the effect of 1,25(OH)_2_D3 on AMP and IL-8 levels in the whole group by pooling the data from the asthma patients and healthy controls. We calculated the median change (difference between the value on day 7 and day 1 of the treatment period) and used a Wilcoxon signed rank test to evaluate if the difference in treatment period was significant. After treatment with active vitamin D, HNP1-3 increased significantly in the whole group (median change after placebo: -151 ng/ml; after 1,25(OH)_2_D3: 1291 ng/ml; p = 0.04) and also IL-8 increased significantly (median change after placebo; -5ng/ml; after 1,25(OH)_2_D3 9 ng/ml; p = 0.02), whereas no significant increases were observed for the levels of the other AMPs shown in Fig 4.

## Discussion

Analysis of nasal secretions showed that the levels of the antimicrobial peptides (AMPs) HNP1-3 and LCN2 were significantly lower in allergic asthmatics compared to non-allergic controls, whereas hCAP18/LL-37 and SLPI did not differ. Plasma hCAP18/LL-37 was correlated with 25(OH)D3 in the total group of participants at baseline. Furthermore in the total group of participants, treatment with 1,25(OH)_2_D3 caused a small but significant increase in nasal HNP1-3. When we analyzed asthmatic and healthy participants separately, 1,25(OH)_2_ vitamin D3 treatment was associated with an non-significant increase in HNP1-3 in the group of asthmatics, whereas in healthy controls the reverse was observed. LCN2 levels also increased to a limited extent, but these effects did not reach statistical significance; hCAP18/LL-37 and SLPI showed no differences. We found IL-8 levels at baseline to be significantly higher in healthy controls. After 1,25(OH)_2_D3 treatment IL-8 levels significantly increased in the total group of participants. We conclude that levels of HNP1-3 and LCN2 are lower in nasal secretions in asthmatics, and that treatment with active 1,25(OH)_2_D3 causes only a small increase in HNP1-3.

Our observation that nasal HNP1-3 and LCN2 were significantly lower in the asthma patients is in line with observations in previous studies showing lower AMP levels in allergy[7]. This difference was noted, despite the fact the number of participants was limited. These lower levels may in part be explained by the ability of Th2 cytokines to decrease epithelial expression of AMPs[6,10], but this does not explain the lower levels of neutrophil-derived HNP1-3. Other studies reported higher sputum LCN2 levels in asthma patients compared to controls[23]. and higher LCN2 in nasal secretions during the pollen season in asthma compared to controls[24]. Furthermore higher HNP1-3 levels in asthma patients were observed after exposure to rhinovirus[25]. These different results may be explained by different patient groups, disease activity, the analysis of sputum or BAL fluid versus nasal secretions, and the effect of allergen or viral exposure[24,25]. A previous study also showed that sputum LL-37 was lower in asthma compared to controls[10], whereas we did not find significant differences for hCAP18/LL-37 in nasal secretions. It needs to be recognized that detection errors may also contribute to differences in outcomes between studies, since especially LL-37 is known to stick to anionic substances such as mucins[26] that are abundantly present in e.g. nasal secretions. Although the observed relative deficiency of AMPs was not unexpected, the observed lower IL-8 levels in asthmatics were not in line with previous studies that showed higher levels of this chemokine in asthma[10]. Since patients did not use inhaled or nasal steroids, such treatment is not likely to have interfered with our results.

How can we interpret and explain the effects of vitamin D on AMPs and IL-8 levels in nasal secretions? Vitamin D has been shown to increase expression of LL-37 *in vivo* [14,15] and epithelial expression of LL-37 and LCN2 *in vitro*[11,12]. We found a correlation between plasma hCAP18/LL-37 and serum 25(OH)D3 at baseline and thus confirmed previous findings in other patient populations [27]. Furthermore, we found a small, but significant increase of HNP1-3 after 1,25(OH)_2_D3 treatment in the whole group. The interpretation of the observation that HNP1-3 increases with vitamin D treatment in the whole group is complicated by the observation that vitamin D treatment had opposite effects on HNP1-3 in asthmatics and healthy controls. Whereas treatment appeared to cause a non significant increase in the asthmatics, in healthy controls placebo caused a stronger increase than vitamin D treatment suggesting large variations in HNP1-3 levels. Therefore these results on HNP1-3 need to be interpreted with caution. The vitamin D-induced increase in LCN2 was not significant and we did not observe an increase of LL-37 and SLPI after vitamin D treatment. Interestingly, there was a significant effect of vitamin D treatment on HNP1-3 which is only expressed by neutrophils, and a non significant increase in LCN2 that is expressed by both neutrophils and epithelial cells. In contrast, SLPI that is mainly expressed by epithelial cells was not affected by treatment. Previous studies have shown that topical application of vitamin D may increase local LL-37 expression in skin[28], and oral administration may be associated with an increased number of hCAP18/LL-37 expressing neutrophils in the circulation[15]. In line with our findings, oral administration of vitamin D during 12 weeks to cystic fibrosis patients did not affect circulating levels of hCAP18/LL-37 and LCN2[29]. Possibly, the number of patients in the present study was too low to observe an effect of vitamin D. Alternatively, despite the observed increases of epithelial LL-37 by vitamin D observed in *in vitro* studies, the contribution of vitamin D to respiratory epithelial cell-derived hCAP18/LL-37 levels in secretions *in vivo* may only be limited.

The higher IL-8 levels observed following vitamin D treatment are unexpected. Previous studies have shown that vitamin D decreases poly(I:C)-induced IL-8 release by bronchial epithelial cells[30,31]. Possibly the increased levels of HNP1-3 following vitamin D treatment have contributed to this increased IL-8, since HNP1-3 has been shown to increase IL-8 production in airway epithelial cells [32].

A strength of this study is the comparison of multiple AMPs in airway secretions from allergic asthmatics and healthy controls, and the use of minimally manipulated nasal fluid instead of nasal lavage. Furthermore, the use of active vitamin D treatment for the intervention is another strength of the study, because it prevents the bias introduced by differences in local conversion of 25(OH)D to active vitamin D. The limited number of participants is a weakness of our study. However, because we used a placebo-controlled cross-over design, we were able to analyze within subject treatment effects. Nevertheless, we only found a small but significant increase in HNP1-3 which may be explained by the large range of AMP levels and the small group size. Furthermore, four of our patients did not show an increase of 1,25(OH)_2_D3 after treatment. This may indicate a compliance problem, which thus could have weakened our results. However, we did collect the used medication strips to assess compliance, and did not notice a problem. In addition, it needs to be noted that whereas treatment did result in a significant increase in circulating 1,25(OH)_2_D3 levels, this increase was unexpectedly not correlated with the observed increase in nasal AMPs. At present it is not clear whether this is due to the relatively small subgroups studied, variability in the measurements of AMPs and 1,25(OH)_2_D3, or to the absence of such a correlation because 1,25(OH)_2_D3 acts locally. Furthermore, because of the small sample size we did not study polymorphisms in the vitamin D receptor or vitamin D binding protein that may have contributed to our results. Finally, our study does not provide information on AMP expression in the lower airways. The induced sputum samples that were collected during the study were of insufficient quality to allow a reliable analysis.

We conclude that levels of selected AMPs are lower in nasal secretions in asthmatics, and treatment with active vitamin D increases the levels only to a limited extent when analyzed in the combined group of healthy controls and asthmatics. Whether this increase is biologically and clinically relevant is unclear at the present, and requires additional studies in a larger population Our study suggests that allergic inflammation contributes to impaired antimicrobial peptides levels and vitamin D has only very limited effects on these levels. Possibly the effect of vitamin D is more marked in asthma patients with a severe vitamin D deficiency, as has also been suggested in COPD[33]. To our knowledge this is the first study to evaluate the effect of active vitamin D administration in patients with asthma on AMP expression. These results contribute to our understanding of the effects of allergic asthma on the innate immune system and release of different AMPs, and of how vitamin D can modulate immune functions in these patients.

## Supporting Information

S1 Fig Baseline levels of serum 25(OH)D3 in asthma patients (A; n = 19) and healthy controls (B; n = 23). (TIF)
