# Supplementary figures and images for: Nasal Levels of Antimicrobial Peptides in Allergic Asthma Patients and Healthy Controls: Differences and Effect of a Short 1,25(OH)2 Vitamin D3 Treatment
Source: PLoS One. 2015 Nov 6;10(11):e0140986. doi: 10.1371/journal.pone.0140986 (PMC4636236; doi:10.1371/journal.pone.0140986)

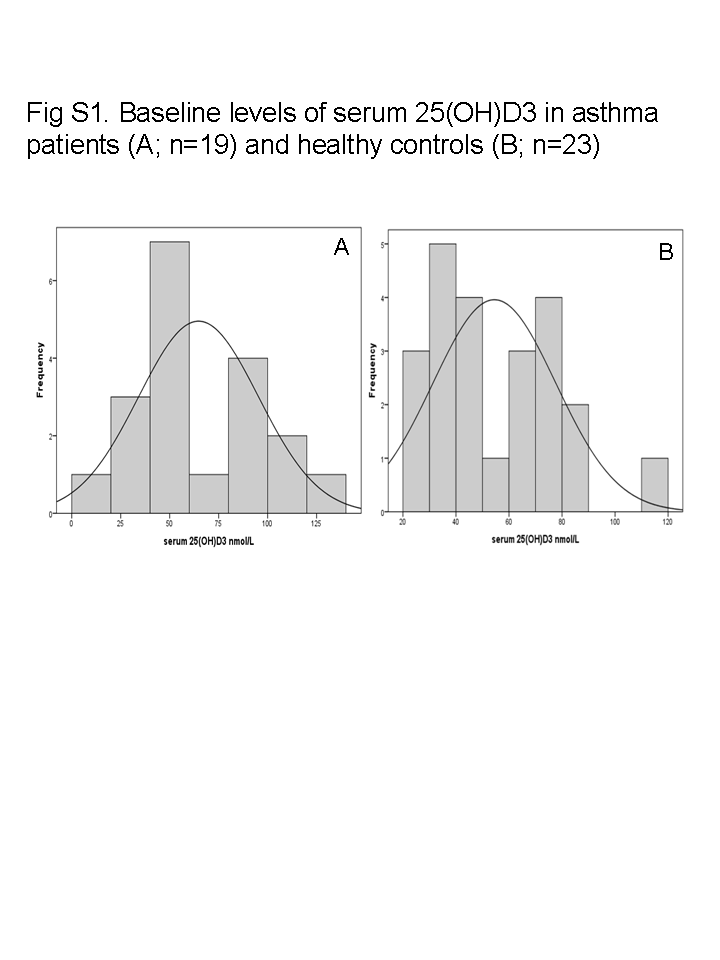

Supplement: S1 Fig — (TIF) [file pone.0140986.s001.tif]
